# Effect of heart rate correction on pre- and post-exercise heart rate variability to predict risk of mortality—an experimental study on the FINCAVAS cohort

**DOI:** 10.3389/fphys.2014.00208

**Published:** 2014-06-03

**Authors:** Paruthi Pradhapan, Mika P. Tarvainen, Tuomo Nieminen, Rami Lehtinen, Kjell Nikus, Terho Lehtimäki, Mika Kähönen, Jari Viik

**Affiliations:** ^1^Department of Electronics and Communication Engineering, Tampere University of TechnologyTampere, Finland; ^2^BioMediTechTampere, Finland; ^3^Department of Applied Physics, University of Eastern FinlandKuopio, Finland; ^4^Department of Clinical Physiology and Nuclear Medicine, Kuopio University HospitalKuopio, Finland; ^5^Heart and Lung Centre, Helsinki University Central HospitalHelsinki, Finland; ^6^Tampere Polytechnic, University of Applied SciencesTampere, Finland; ^7^School of Medicine, University of TampereTampere, Finland; ^8^Heart Centre, Department of Cardio-Thoracic Surgery, Tampere University HospitalTampere, Finland; ^9^Fimlab Laboratories, Department of Clinical ChemistryTampere, Finland; ^10^Department of Clinical Physiology, Tampere University HospitalTampere, Finland

**Keywords:** heart rate correction, heart rate variability, receiver operating characteristics, Kaplan-Meier, FINCAVAS

## Abstract

The non-linear inverse relationship between RR-intervals and heart rate (HR) contributes significantly to the heart rate variability (HRV) parameters and their performance in mortality prediction. To determine the level of influence HR exerts over HRV parameters' prognostic power, we studied the predictive performance for different HR levels by applying eight correction procedures, multiplying or dividing HRV parameters by the mean RR-interval (RR_avg_) to the power 0.5–16. Data collected from 1288 patients in The Finnish Cardiovascular Study (FINCAVAS), who satisfied the inclusion criteria, was used for the analyses. HRV parameters (RMSSD, VLF Power and LF Power) were calculated from 2-min segment in the rest phase before exercise and 2-min recovery period immediately after peak exercise. Area under the receiver operating characteristic curve (AUC) was used to determine the predictive performance for each parameter with and without HR corrections in rest and recovery phases. The division of HRV parameters by segment's RR_avg_ to the power 2 (HRV_DIV-2_) showed the highest predictive performance under the rest phase (RMSSD: 0.67/0.66; VLF Power: 0.70/0.62; LF Power: 0.79/0.65; cardiac mortality/non-cardiac mortality) with minimum correlation to HR (*r* = −0.15 to 0.15). In the recovery phase, Kaplan-Meier (KM) survival analysis revealed good risk stratification capacity at HRV_DIV-2_ in both groups (cardiac and non-cardiac mortality). Although higher powers of correction (HRV_DIV-4_and HRV_DIV-8_) improved predictive performance during recovery, they induced an increased positive correlation to HR. Thus, we inferred that predictive capacity of HRV during rest and recovery is augmented when its dependence on HR is weakened by applying appropriate correction procedures.

## Introduction

Heart rate (HR) recovery and heart rate variability (HRV) have been used by researchers for assessing the role of autonomic regulation in predicting all-cause and cardiovascular mortality (Freeman et al., 2006). The prognostic capabilities of HR response to exercise and after exercise have been well-documented (Lauer et al., 1996; Cole et al., 1999; Lipinski et al., 2004; Jouven et al., 2005; Kiviniemi et al., 2011) and reviewed by Freeman et al. (2006). Increased sympathetic and decreased parasympathetic activities have been associated with an enhanced risk of sudden death or the vulnerability to ventricular arrhythmias (Lahiri et al., 2008). Subdued time- and frequency-domain HRV indices have been linked with increased risk of mortality in the Framingham cohort (Tsuji et al., 1994), survivors of acute myocardial infarction (MI) (Kleiger et al., 1990; Kiviniemi et al., 2007) and cardiovascular morbidity and mortality (Zuanetti et al., 1996). However, studies determining the prognostic capacity of exercise induced short-term HRV have been sparse and inconsistent. Leino et al. (2010) concluded that none of the HRV indices were good predictors of mortality during peak exercise or recovery phase. In a study by Dewey et al. (2007), a greater short-term HRV during recovery post exercise was associated with an increased risk for all-cause and cardiovascular mortality. This is in contrast to observations made in resting HRV, which implies higher RR-interval variability is associated with better prognosis (Dekker et al., 2000; Leino et al., 2010).

Nieminen et al. (2007) justified that the non-linear inverse relationship between RR interval and HR could be the cause for misinterpretation when comparing subjects with different HR levels and this has been concurred by other researchers (Chiu et al., 2003; Sacha and Pluta, 2005; Sacha et al., 2005; Virtanen et al., 2007; Bailón et al., 2011). Possible physiological mechanisms involved have also been probed (Perini and Veicteinas, 2003; Goldberger et al., 2006). The non-linear relation between HR and HRV has been addressed by Sacha and Pluta (2008) and correction methods have been suggested to strengthen or weaken the influence of HR (Sacha et al., 2013a; Sacha, 2013). By determining whether decreasing dependence on HR improves the prognostic capacity of HRV, we sought to establish the influence of HR in predicting mortality risk. The aim of our study was to scrutinize these correction techniques and their influence on the predictive capacity of cardiac and non-cardiac mortality in the Finnish Cardiovascular Study (FINCAVAS) cohort.

## Materials and methods

### Patient population and follow-up

A total of 2212 consecutive patients, who were referred by a physician and willing to undergo exercise stress tests at the Tampere University Hospital, were recruited between 2001 and 2004 for FINCAVAS. Informed consent was obtained from all the participants prior to the interview. Measurements were conducted as stipulated in the Declaration of Helsinki and the study protocol was approved by the Ethical Committee of the Hospital District of Pirkanmaa, Finland. In addition to raw electrocardiograph (ECG), descriptive information, medical history and habitual lifestyle of each patient were recorded. More detailed information regarding the patient population and sample size determination is described elsewhere (Nieminen et al., 2006). Of these, 1288 patients satisfied the inclusion criteria for this study with good quality HRV measurements for at least 2 min during rest phase, immediately prior to exercise, and 2 min during post-exercise recovery immediately after maximum effort.

The follow-up data consisted of information related to causes of death and was collected in 2011. The information for the follow-up was obtained from Causes of Death Register and has been shown to be reliable (Pajunen et al., 2005). The follow-up yielded 66 cardiac deaths and 94 non-cardiac deaths, while the remaining 1128 patients constituted the survival group.

### Exercise testing protocol

The prognoses of mortality were analyzed using HRV indices obtained from 2 min segments during rest phase before exercise and 2 min recovery immediately after maximal exercise. Resting ECG was measured in the supine position prior to exercise. The exercise stress test was then performed on a bicycle ergometer with electrical brakes and the Mason-Likar modified lead system (Mason and Likar, 1966) was used for the ECG data acquisition. Initial work load and increments were defined based on patient's age, gender, body mass index (BMI) and physical activity. Starting work load varied between 20 and 30 W and the stepwise increments ranged between 10 and 30 W every minute. ECG and HR were measured continuously during the test. Tests were sign- and symptom-limited with the recommended criteria for termination whereas in the case of post-MI patients, the upper limit for HR was set at 120–130 beats per minute (bpm). The chronotropic response index (CRI), which represents the chronotropic response to exercise, was evaluated as CRI = 100 × (peak HR − resting HR)/(220 − age − resting HR) (Kiviniemi et al., 2011). CRI < 80% was defined as low reserve capacity (Lauer et al., 1996). Measurement during the recovery phase was performed in the sitting position, immediately after exercise.

### HRV measurement

ECG was recorded at a sampling frequency of 500 Hz using CardioSoft exercise ECG system (Version 4.14, GE Healthcare, Freiburg, Germany) and was analyzed using Modified CASE software (GE Healthcare, Freiburg, Germany). After producing the RR-interval tachogram, the data was preprocessed to remove abnormal intervals and artifacts before they were divided into shorter segments based on the stages of rest and recovery. HRV parameters were determined using the Kubios HRV analysis software (Tarvainen et al., 2014). All intervals were resampled using cubic spline interpolation at 4 Hz. Linear and smoothness prior (smoothing parameter, λ = 500) detrending were performed prior to calculating time-domain parameters. The fast Fourier transform (FFT) spectrum was computed with a window width of 240 samples, which corresponds to the length of 1 min segment with 4 Hz resampling rate. A 50% overlapping window was used for longer segments. Mean RR intervals (RR_avg_) were calculated from each segment individually for the HR correction procedure.

Post-exercise recovery is marked by sympathetic withdrawal and parasympathetic reactivation. Sympathetic activation and attenuated parasympathetic recovery are significantly associated with adverse prognosis. The parameters included for examination were chosen based on previous HRV studies on mortality prediction and its outcomes. Of the spectral measures, low frequency (0.04–0.15 Hz, LF) power has been found to increase during exercise in normal subjects and reflects both sympathetic and vagal influences (Malliani et al., 1991). In addition, higher log LF power during recovery significantly predicted increased risk of all-cause and cardiovascular mortality (Dewey et al., 2007). Bigger et al. (1993) demonstrated that spectral measures from short segments (2–15 min) correlated significantly with those computed using 24-h periods. Bernardi et al. (1996) indicated that very low frequency (0.0033–0.04 Hz, VLF) power fluctuations were highly dependent on changes in physical activity, rather than preconceived notion of reflecting autonomic tone and thereby, emphasized the importance of activity as a confounding factor. Therefore, VLF power was evaluated due to its independent risk stratification property for all-cause mortality in patients with acute MI (Bigger et al., 1993). Although high frequency (0.15–0.4 Hz, HF) power has been frequently used to measure parasympathetic tone in resting HRV, interpreting values during recovery after exercise is complicated due to tonic autonomic activity and residual adrenergic activity (Dewey et al., 2007). Goldberger et al. (2006) demonstrated that short-term (as small as 30 s windows) root mean squared difference of successive RR intervals (RMSSD), which represents high frequency variations in HR, is adequate for measuring parasympathetic reactivation in recovery phase.

### HR correction

As described by Sacha et al. (2013a), the HRV dependence on HR is strengthened or weakened by multiplying or dividing the HRV indices by the corresponding segment's RR_avg_, respectively. In addition to normal determination of HRV indices, eight other classes for the indices were assessed in this study: HRV_MUL-0.5_—multiplying HRV indices by RR_avg_ to the power 0.5; HRV_MUL-2_—multiplying HRV indices by RR_avg_ to the power 2; HRV_MUL-4_—multiplying HRV indices by RR_avg_ to the power 4; HRV_DIV-0.5_—dividing HRV indices by RR_avg_ to the power 0.5; HRV_DIV-2_—dividing HRV indices by RR_avg_ to the power 2; HRV_DIV-4_—dividing HRV indices by RR_avg_ to the power 4; HRV_DIV-8_—dividing HRV indices by RR_avg_ to the power 8; and HRV_DIV-16_—dividing HRV indices by RR_avg_ to the power 16. With these classes, different levels of dependence/independence to HR were attained and can be considered significant in determining the contribution of HR in prognosis of cardiac and non-cardiac mortalities.

### Statistical analyses

The relative risks for cardiac and non-cardiac mortality were assessed for individual characteristics, clinical condition and medication using univariate Cox models. The measure of the predictive power for different HR correction methods for each segment was computed using area under the receiver operating characteristics (ROC) curve. Spearman's rank correlation was performed to determine the degree of correspondence to HR. The cut-off points for Kaplan-Meier (KM) survival analyses were defined from the ROC analyses for each segment. The point of highest overall predictive performance (average of sensitivity and specificity) was chosen as the cut-off to distinguish mortality and survival groups based on HRV observed in the patient population. It has to be noted that these cut-off points were not optimized in order to preserve uniformity during comparisons. The Log-rank chi-square estimates were then used to evaluate the significance of the correction methods based on this classification.

## Results

During the follow-up of the patients who satisfied the inclusion criteria, 66 cardiac deaths were recorded, which included 31 sudden cardiac deaths, with a mean follow-up time of 54 months (min: 4.8 days; max: 99.5 months). 94 patients died of non-cardiovascular causes between 1.2 and 110.7 months of follow-up (mean: 60.2 months). The baseline characteristics, clinical conditions and medications used by patients who suffered cardiac and non-cardiac deaths are listed in Table 1.

**Table 1:** **Baseline characteristics of the study population, classified into survival, cardiac, and non-cardiac mortality groups**.

	**Survival group (*N* = 1128)**	**Mortality group (*N* = 160)**		
		**Cardiac mortality (*N* = 66)**	**Non-cardiac mortality (*N* = 94)**	***p*-value**
**INDIVIDUAL FACTORS**				
Age (years)	54.3 ± 12.6	61.6 ± 10.9	64.1 ± 10.5	0.145
Gender (males, %)	699 (62.0)	42 (78.8)	58 (61.7)	0.020
BMI	27.4 ± 4.5	28.9 ± 4.7	27.0 ± 3.9	0.004
Smoking (yes, %)	317 (28.1)	20 (30.3)	32 (34.0)	0.622
CRI (%)	82.8 ± 24.4	62.3 ± 30.1	73.5 ± 29.8	0.021
Resting heart rate (bpm)	63.3 ± 11.3	64.8 ± 13. 9	64.5 ± 12.5	0.656
SAP at rest (mmHg)	135.8 ± 18.5	134.4 ± 21.1	136.3 ± 20.2	0.563
DAP at rest (mmHg)	79.7 ± 9.6	78.1 ± 9.9	77.3 ± 12.2	0.675
Maximum heart rate (bpm)	149.1 ± 25.7	125.6 ± 27.2	132.1 ± 26.5	0.106
SAP peak exercise (mmHg)	196.2 ± 28.6	179.7 ± 32.9	184.8 ± 27.8	0.296
DAP peak exercise (mmHg)	92.4 ± 12.3	88.2 ± 12.2	87.7 ± 13.4	0.813
**CLINICAL CONDITION**				
CHD (yes, %)	360 (31.9)	30 (45.5)	32 (34.0)	0.146
MI (yes, %)	226 (20.0)	24 (36.4)	22 (23.4)	0.075
Diabetes (yes, %)	128 (11.3)	15 (22.7)	14 (14.9)	0.208
**MEDICATION**				
ACE inhibitors (yes, %)	235 (20.8)	26 (39.4)	21 (22.3)	0.020
Beta blockers (yes, %)	639 (56.6)	56 (84.8)	70 (74.5)	0.116
Calcium channel blockers (yes, %)	179 (15.9)	17 (25.8)	19 (20.2)	0.412
Diuretics (yes, %)	180 (16.0)	20 (30.3)	28 (29.8)	0.945
Lipid medication (yes, %)	443 (39.3)	39 (59.1)	44 (46.8)	0.127
Nitrates (yes, %)	357 (31.6)	32 (48.5)	44 (46.8)	0.208

Values are expressed as Mean ± SD or number of subjects (%). BMI, body mass index; CRI, chronotropic response index; SAP, systolic arterial pressure; DAP, diastolic arterial pressure; CHD, coronary heart disease; MI, myocardial infarction.

The univariate Cox regression results for various factors associated with cardiac and non-cardiac mortality are presented in Table 2. The relative risk (RR) of cardiac death was significantly higher in males [*RR* = 2.27, 95% confidence interval (CI) = 1.26–4.09, *p* < 0.05]. Age ≥ 60 years was a risk factor for cardiac (*RR* = 2.33, 95% *CI* = 1.43–3.80, *p* < 0.001) and non-cardiac (*RR* = 3.01, 95% *CI* = 1.98–4.58, *p* < 0.001) mortality. Clinical conditions were significantly associated with risk of cardiac death. Medication such as ACE inhibitors (*RR* = 2.37, 95% *CI* = 1.45–3.89) and beta blockers (*RR* = 3.95, 95% *CI* = 2.02–7.75) were significantly associated with increased risk of cardiac mortality (*p* < 0.001).

**Table 2:** **Association of individual factors, clinical conditions and medication to cardiac and non-cardiac mortality based on univariate Cox regression**.

	**Cardiac mortality (*N* = 66)**		**Non-cardiac mortality (*N* = 94)**	
	**RR (95% CI)**	***p*-value**	**RR (95% CI)**	***p*-value**
**INDIVIDUAL FACTORS**				
Age ≥ 60 years	2.33 (1.43–3.80)	< 0.001	3.01 (1.98 – 4.58)	< 0.001
Gender (male)	2.27 (1.26–4.09)	< 0.05	0.98 (0.65–1.48)	0.91
BMI ≥ 25	1.40 (0.76–2.56)	0.001	1.08 (0.68–1.73)	0.47
Smoking (yes)	1.10 (0.65–1.85)	0.13	1.29 (0.84–1.98)	0.21
CRI ≤ 80%	3.95 (2.25–6.93)	< 0.001	2.02 (1.33–3.08)	< 0.001
CRI ≤ 39%	4.98 (2.76–8.99)	< 0.001	2.63 (1.43–4.82)	< 0.001
HR_rest_ ≥ 80 bpm	0.59 (0.32–1.06)	0.08	0.70 (0.44–1.17)	0.13
HR_max_ ≤ 120 bpm	3.69 (2.27–6.00)	< 0.001	2.12 (1.37–3.27)	< 0.001
**CLINICAL CONDITIONS**				
CHD (yes)	1.72 (1.06–2.78)	< 0.05	1.05 (0.68–1.60)	0.84
MI (yes)	2.18 (1.32–3.60)	< 0.001	1.16 (0.72–1.86)	0.55
Diabetes (yes)	2.16 (1.21–3.84)	< 0.05	1.29 (0.73–2.27)	0.38
**MEDICATION**				
ACE inhibitors (yes)	2.37 (1.45–3.89)	< 0.001	1.06 (0.65–1.73)	0.81
Beta blockers (yes)	3.95 (2.02–7.75)	< 0.001	2.03 (1.28–3.23)	< 0.05
Calcium channel blockers (yes)	1.76 (1.01–3.05)	< 0.05	1.29 (0.78–2.13)	0.33
Diuretics (yes)	2.09 (1.24–3.54)	< 0.05	2.09 (1.34–3.25)	< 0.05
Lipid medication (yes)	2.11 (1.29–3.45)	< 0.05	1.29 (0.86–1.93)	0.22
Nitrates (yes)	1.87 (1.16–3.03)	< 0.05	1.70 (1.13–2.55)	< 0.05

CI, confidence interval; RR, relative risk; BMI, body mass index; CRI, chronotropic response index; HR_rest_, resting heart rate; HR_max_, maximum heart rate achieved during peak exercise; CHD, coronary heart disease; MI, myocardial infarction.

**Figure 1:**
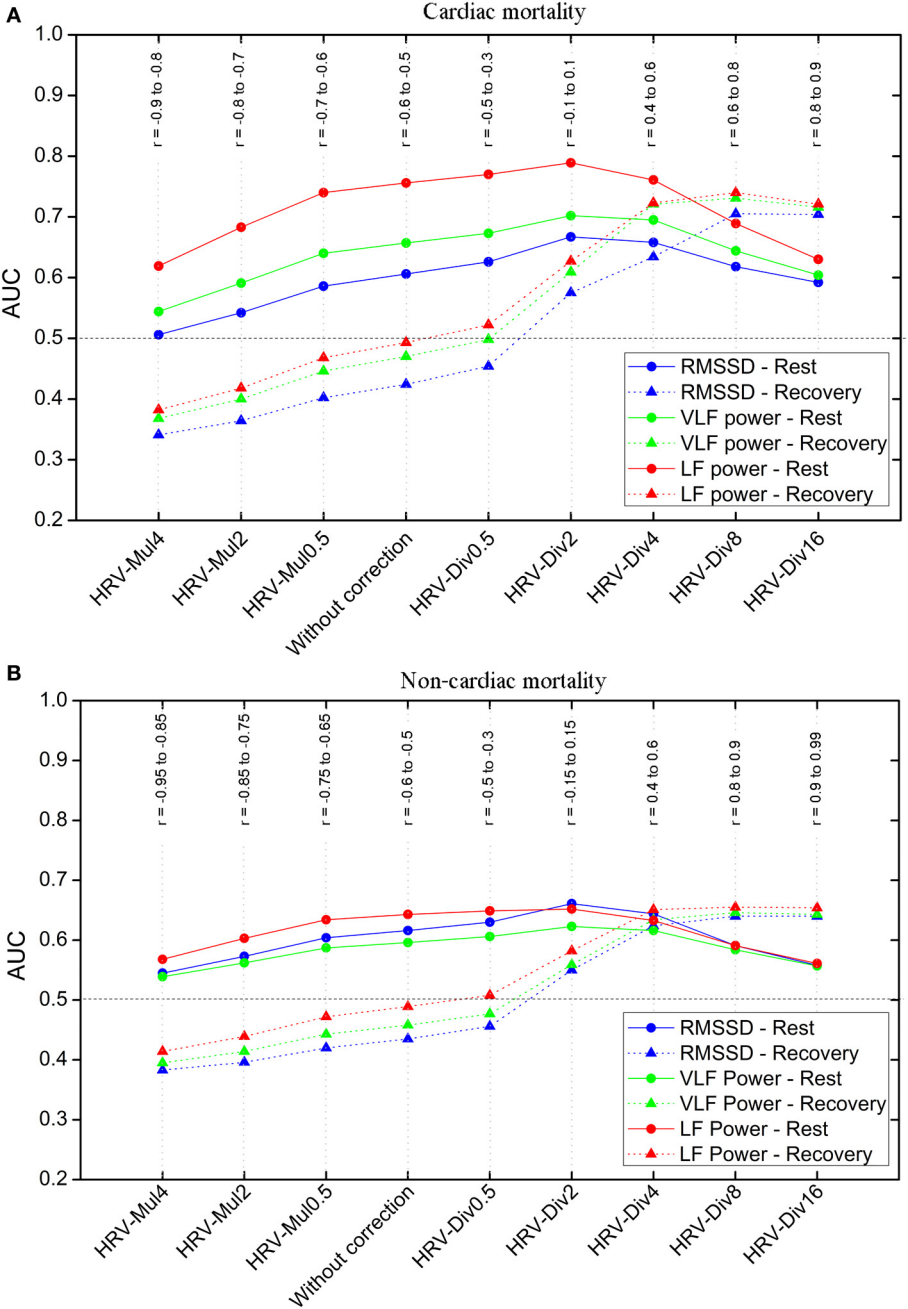
**Predictive performance of heart rate variability (HRV) parameters for: (A) cardiac mortality and (B) non-cardiac mortality groups**. Area under the receiver operating characteristics curves (AUC) and correlation coefficients (r), between HRV parameters and HR, for different correction methods during rest and recovery after exercise. AUC > 0.5 indicates that higher heart rate variability (HRV) is associated with better prognosis and AUC < 0.5 indicates higher HRV is associated with worse prognosis.

The area under the ROC curve (AUC) for HR was found to be 0.57/0.70 (rest/recovery) for cardiac mortality and 0.53/0.64 for non-cardiac mortality, implying that HR is a better predictor during recovery than during rest phase. The AUC for RMSSD, VLF and LF power, calculated under different correction methods during rest and recovery phases are presented in Figures 1A,B. Correlation with HR (*r*, presented in Figure 1) indicated increasing dependence or independence of HRV to HR, based on the method of correction used. AUC > 0.5 suggested that higher HRV are indicative of better prognosis. HRV_DIV-2_, which revealed minimum correlation to HR, was the best predictor for both outcomes (cardiac and non-cardiac mortality) in the rest phase. However, during recovery, higher standard HRV (i.e., HRV without correction) was associated with worse prognosis (AUC < 0.5), as seen in Figure 1. In addition, similar associations were observed for HRV parameters multiplied by different powers of RR_avg_ (HRV_MUL-0.5_, HRV_MUL-2, and_ HRV_MUL-4_). Conversely, after division by higher powers of RR_avg_ (i.e., for HRV_DIV-2_, HRV_DIV-4_, HRV_DIV-8_, and HRV_DIV-16_), higher HRV was associated with better prognosis (AUC > 0.5). Though higher orders of correction resulted in better predictive capacity, it also induced moderate/strong positive correlation to HR (in the case of HRV_DIV-4_, HRV_DIV-8_, and HRV_DIV-16_).

**Figure 2:**
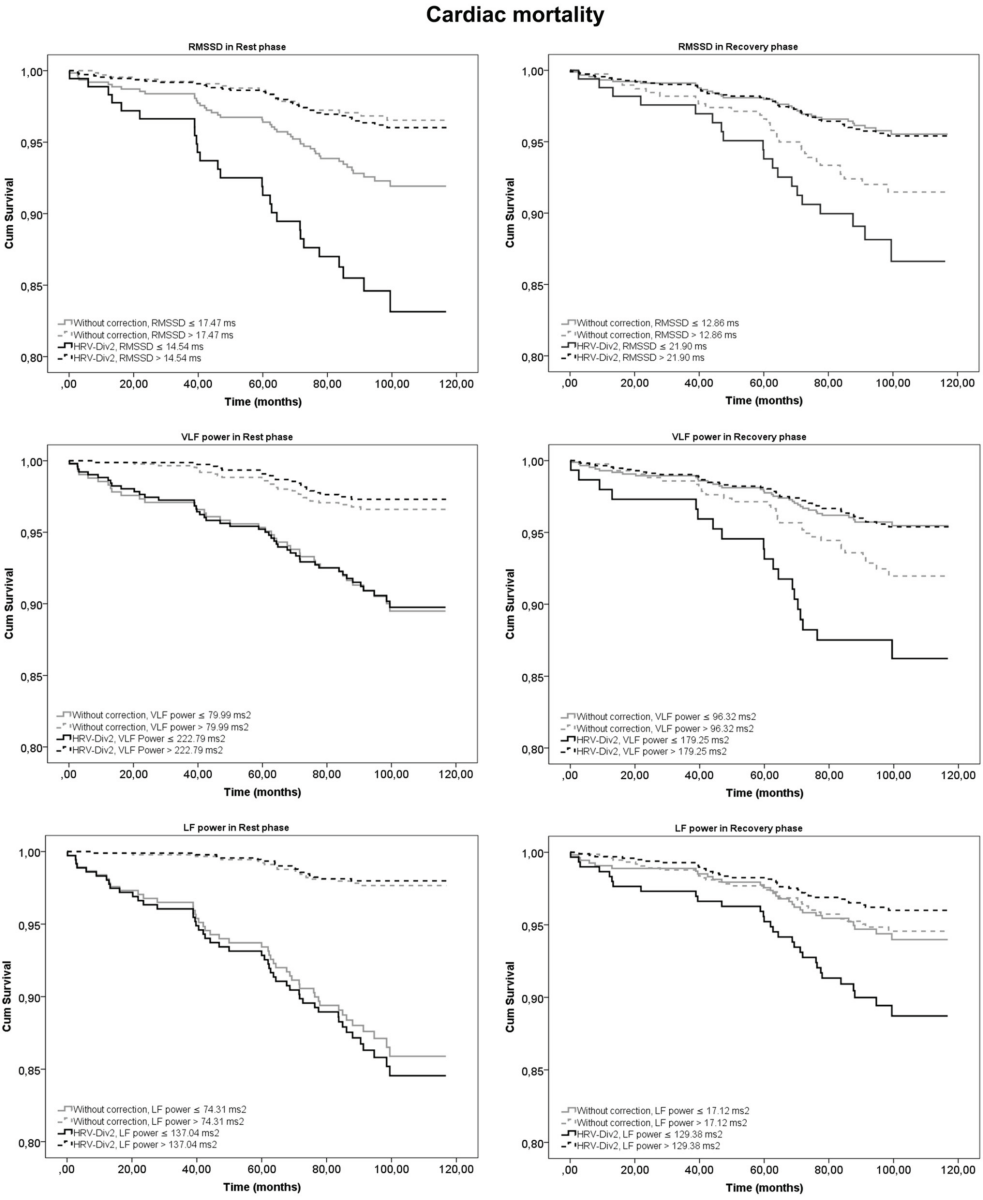
**Kaplan-Meier (KM) survival curves for prediction of cardiac mortality using heart rate variability (HRV) parameters at rest and recovery after exercise**. Curves in gray represent HRV indices without correction and in black indicate the survival estimates for the best correction with minimum dependence on heart rate (HRV_DIV-2_).

**Figure 3:**
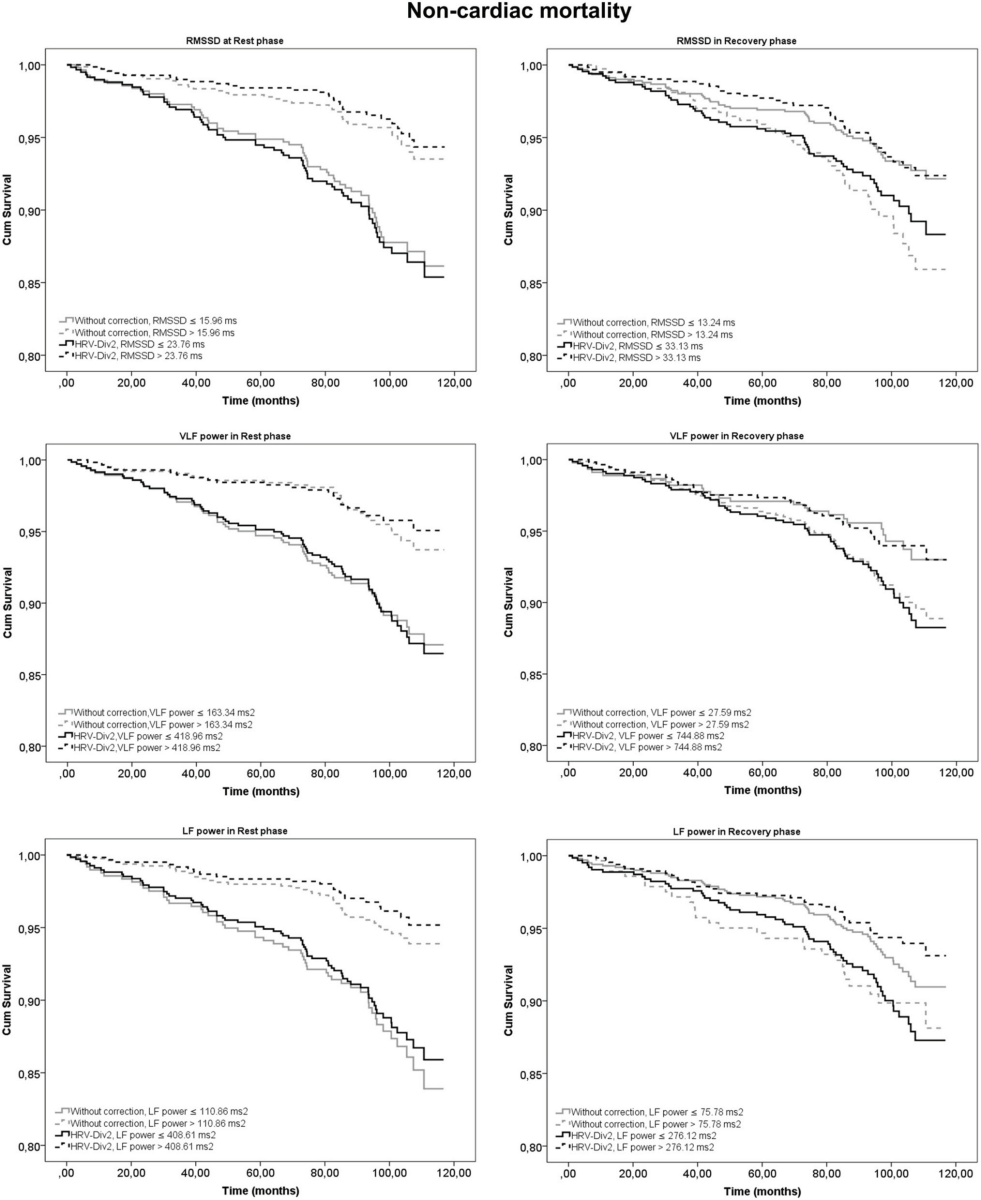
**Kaplan-Meier (KM) survival curves for prediction of non-cardiac mortality using heart rate variability (HRV) parameters at rest and recovery after exercise**. Curves in gray represent HRV indices without correction and in black indicate the survival estimates for the best correction with minimum dependence on heart rate (HRV_DIV-2_).

These results were further corroborated by KM survival analysis. Log-rank estimates at different degrees of correction for both cardiac and non-cardiac mortality are presented in Table 3. HRV_MUL-4_ and HRV_DIV-16_ were excluded due to their very strong correlation to HR. Mortality prediction was most significant for HRV_DIV-2_ in the rest phase. During recovery, the division of HRV by higher powers of RR_avg_ resulted in better risk stratification for cardiac and non-cardiac deaths. Although HRV_DIV-4_ and HRV_DIV-8_ exhibited better predictive powers during recovery, the HRV indices exhibited strong positive correlation to HR (*r* = 0.6 to 0.9 across both groups, as shown in Figure 1) at these correction levels. On the contrary, HRV_DIV-2_ was a good predictor of cardiac (*p* < 0.001) and non-cardiac (*p* < 0.05) mortality during recovery, with minimum influence of HR (*r* = −0.15 to 0.15). Figures 2, 3 represent the survival curves for HRV_DIV-2_ during rest and recovery.

**Table 3:** **Chi-square values for Kaplan-Meier analyses under different heart rate correction methods for cardiac and non-cardiac mortality**.

**Parameter**	**HRV_MUL-2_**	**Without correction**	**HRV_DIV-2_**	**HRV_DIV-4_**	**HRV_DIV-8_**
**CARDIAC MORTALITY**					
**Two minute resting period prior to exercise**					
RMSSD	14.10^**^	11.36^**^	43.47^**^	25.22^**^	11.37^**^
VLF power	9.90^*^	21.43^**^	27.56^**^	27.84^**^	15.56^**^
LF power	33.84^**^	61.65^**^	75.37^**^	50.60^**^	25.38^**^
**Two minute recovery period post exercise**					
RMSSD	15.88^**^	7.93^**^	16.98^**^	30.77^**^	35.84^**^
VLF power	13.56^**^	4.81^**^	21.38^**^	48.48^**^	42.57^**^
LF power	5.50^*^	12.72^**^	20.09^**^	41.77^**^	52.71^**^
**NON-CARDIAC MORTALITY**					
**Two minute resting period prior to exercise**					
RMSSD	8.97^*^	16.82^**^	26.64^**^	21.46^**^	10.09^**^
VLF power	7.63^*^	15.54^**^	19.16^**^	19.05^**^	10.44^**^
LF power	16.17^**^	21.24^**^	24.13^**^	17.73^**^	12.46^**^
**Two minute recovery period post exercise**					
RMSSD	18.60^**^	7.83^*^	4.59^*^	16.09^**^	21.21^**^
VLF power	9.56^*^	3.95^*^	5.08^*^	19.22^**^	29.24^**^
LF power	4.43^*^	2.49	8.61^*^	28.39^**^	26.01^**^

Significance is denoted by

* p < 0.05 and

** p < 0.001.

## Discussion

The HRV indices computed from RR-interval measurements correlated with HR as a result of the non-linear relationship between the RR-interval and instantaneous HR (Chiu et al., 2003; Sacha and Pluta, 2005; Sacha et al., 2005; Nieminen et al., 2007; Virtanen et al., 2007; Bailón et al., 2011). Higher variability during rest and lower variability during recovery were associated with better prognosis, and this corresponds to observations made by Dewey et al. (2007). Our results indicate that the predictive capacity of HRV at rest was highest when the correlation to HR was minimum (HRV_DIV-2_, *r* = −0.15 to 0.15), suggesting that exclusion of HR influence on resting HRV improved prognostic capacity for cardiac and non-cardiac mortality. Since HR is a poor predictor at rest, removal of HR influence perchance resulted in improved prognostic capacity. On the contrary, HR during recovery phase exhibited significant risk stratification for both outcomes. Thus, increasing HRV's dependence on HR enhanced its predictive capacity (observed in HRV_DIV-4_ and HRV_DIV-8_). However, higher degrees of correction produced moderate/strong positive correlation to HR, similar to observations made by Sacha et al. (2013c), Sacha (2014). To attain true independence, the correction technique that yields HRV least influenced by HR, needs to be identified. In our study, HRV_DIV-2_ demonstrated improvement in predictability of mortality risk during recovery phase with minimum dependence on HR.

However, conclusive evidence could not be established to distinguish between cardiac and non-cardiac related deaths. This is in contrast to findings by Sacha et al. (2013b), who suggested that increasing the HRV dependence on HR resulted in greater predictive ability for cardiac death and increasing its independence indicated greater predictive power for non-cardiac death. One possible explanation could be that the study population analyzed by Sacha and coworkers comprised only post-MI patients whereas the current study included more heterogeneous patient material.

This study suffered certain limitations. First, the risk factors for individual, clinical conditions and medication were not modeled to determine their contribution toward mortality prediction. By including these variables to the analyses, a more definite conclusion on the cause of mortality could have been established. Second, the patients were not controlled for the type of medication prescribed. The effects of beta blockers and nitrates have been known to affect HR, which could have an effect on the results of HR correction. However, the purpose of the current study was to evaluate the effects of HR correction methods in mortality prediction and therefore, these issues need to be considered in future studies.

## Conclusion

The findings of this study indicate that the predictive power of HRV parameters for both cardiac and non-cardiac mortality is augmented when its dependence on HR is weakened during rest and recovery. In addition, when HR is a good predictor, increasing HRV's dependence on HR further enhances the risk stratification for both modes of death.

## Author contributions

The study was conceptualized by Tuomo Nieminen, Kjell Nikus, Terho Lehtimäki, Mika Kähönen, and Jari Viik. Data acquisition and analysis was performed by Paruthi Pradhapan, Mika P. Tarvainen, Rami Lehtinen, and Jari Viik. All authors contributed equally in drafting and revising the manuscript.

### Conflict of interest statement

The authors declare that the research was conducted in the absence of any commercial or financial relationships that could be construed as a potential conflict of interest.
